# Specific cognitive dysfunctioning and vulnerability to specific psychopathology: A descriptive study on intellectual developmental disorder (intellectual disability)

**DOI:** 10.1192/j.eurpsy.2023.366

**Published:** 2023-07-19

**Authors:** J. Santambrogio, S. Terrevazzi, M. Danese, C. Boldoni, M. Calascibetta, E. Cudazzo, C. Lucca, V. Viganò, G. Minazzi, E. Francia, A. Santarone, L. De Carolis, A. Bianco, A. Hassiotis, M. O. Bertelli, M. Clerici

**Affiliations:** ^1^Casa San Paolo, Adele Bonolis AS.FRA. Foundation, Vedano al Lambro; ^2^Medicine and Surgery, University of Milano-Bicocca, Monza; ^3^Presidio Corberi; ^4^Direction UOC Disabilità; ^5^RSD Papa Giovanni XXIII, ASST Brianza, Limbiate; ^6^Casa Iris; ^7^Clinical Direction, Adele Bonolis AS.FRA. Foundation, Vedano al Lambro; ^8^DEMS, University of Milano-Bicocca, Milano; ^9^CREA, San Sebastiano Foundation, Firenze, Italy; ^10^Division of Psychiatry, UCL, London, United Kingdom; ^11^Department of Mental Health and Addiction, ASST Monza, Monza, Italy

## Abstract

**Introduction:**

Intellectual Developmental Disorder (IDD) is diagnosed with cognitive and adaptive behaviour evaluations. There is increasing evidence of a high prevalence of psychiatric disorders comorbid with IDD. The relationship between specific cognitive dysfunctions and psychiatric vulnerability may provide the basis for a paradigm shift from “intellectually below average IQ” to “neuropsychological characterization”.

**Objectives:**

1) reassessing an IDD sample in cognitive profile and psychiatric comorbidities 2) investigating the correlations between specific cognitive dysfunctions and specific psychiatric diagnoses in IDD.

**Methods:**

120 individuals with IDD from 3 Italian facilities were consecutively evaluated, one group with mild IDD, using WAIS-IV or Leiter-3, TMT, Stroop and TOL tests, after which a professional caregiver did individual interviews (Vineland Adaptive Behavior Scale-II, SPAIDD-G, and STA-DI) to evaluate the patient adaptive behaviour, psychiatric comorbidities and presence of ASD. The second group (more severe IDD), was evaluated only with professional caregiver assessment tools.

**Results:**

90 males and 30 females, mean age 57 years, institutionalized for a mean period of 36.44 years. 52% had no education, 19% a middle school diploma. IDD diagnoses: borderline 3%, mild 16%, moderate 11%, moderate-severe 4%, severe 59%, profound 0%.11% comorbid ASD diagnosis, 29% with ASD after diagnostic re-assessment (STA-DI). 89% physical comorbidities, 58% psychiatric comorbidities, 56% psychoses (Fig. 1). Psychiatric comorbidities re-assessment (SPAIDD-G) identified a significant number of disorders (Fig. 2), despite the medical records showed a low prevalence of psychiatric diagnoses. The consistent quantity of psychotropic drugs prescribed in the sample, possibly reflects the real prevalence of psychopathology. Pearson correlations (p<0.05). WAIS-IV and SPAIDD-G (N=29): *Verbal Comprehension Index* correlates with anxiety disorder and impulse control disorder; *Perceptual Reasoning Index* correlates with nutrition/feeding disorder; *Processing Speed Index* correlates with nutrition/feeding disorder and sexual disorder; *IQ* correlates with ASD, nutrition/feeding, anxiety, sexual disorders. Leiter-3 and SPAIDD-G (N=14): *Form Completion* and *non-verbal IQ* correlate with OCD negatively.

**Image:**

**Figure:**
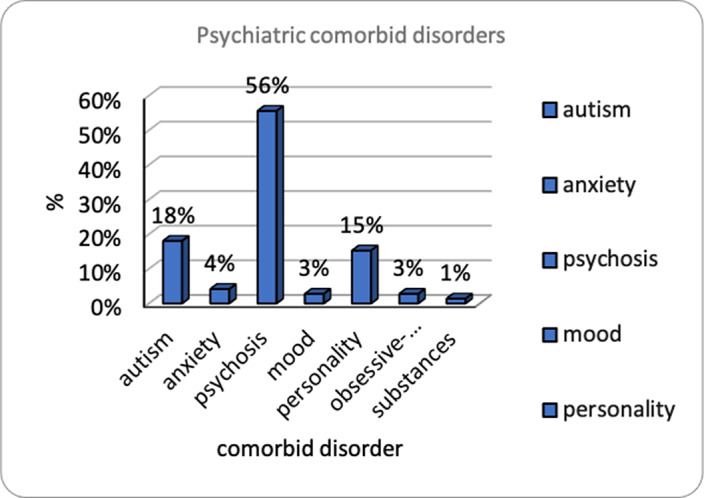

**Image 2:**

**Figure:**
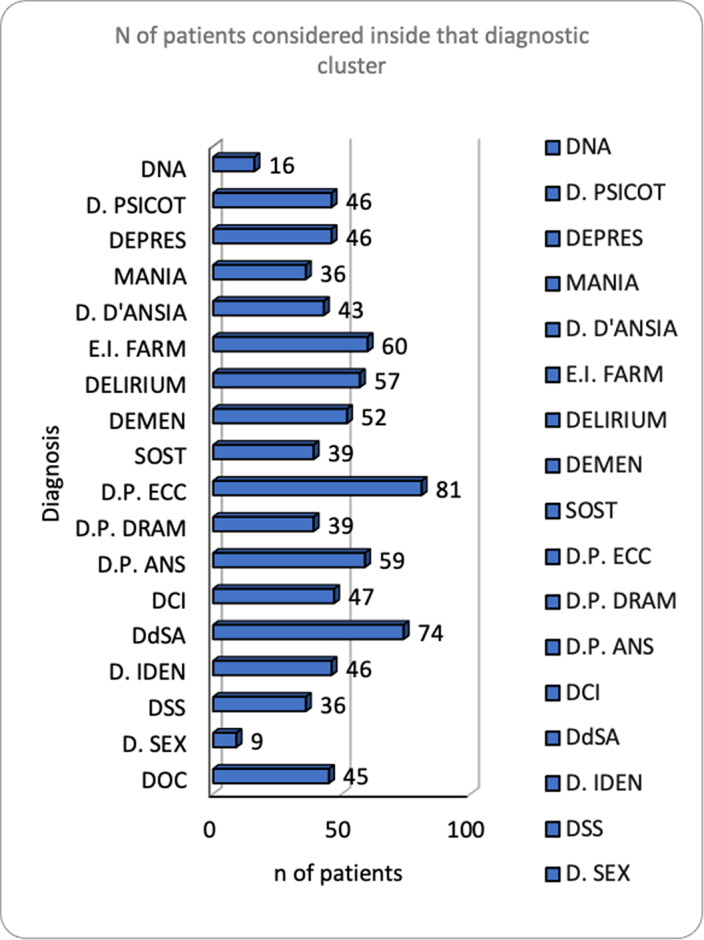

**Conclusions:**

In conclusion, the SPAIDD-G evaluations revealed a greater prevalence of psychopathology than reported in the medical records. Using psychopathological screening tools can improve the diagnostic process in residential facilities for IDD cases. Pearson’s analyses revealed the need to further investigate the correlation between cognitive dysfunctions and psychopathological vulnerability, studying intelligence as a multi-component model and identifying specific behavioural and cognitive phenotypes in IDD cases.

**Disclosure of Interest:**

None Declared

